# The occupational roles of nurses and midwives in the UK: an analysis of the Nursing and Midwifery Council-census England and Wales 2021 data linkage study

**DOI:** 10.23889/ijpds.v9i5.2748

**Published:** 2024-09-18

**Authors:** Michelle Jamieson, Jan Savinc, Iain Atherton

**Affiliations:** 1 Edinburgh Napier University; 2 Scottish Centre for Administrative Data Research

## Objective

The nursing and midwifery professions are facing challenges to recruitment and retention. Understanding the occupational choices of people registered as nurses and midwives can inform policy as to the potential of initiatives to encourage registrants back to practice. This study thus aims to ascertain the occupational roles taken by nurses and midwives in the UK in 2021 and the drivers behind choosing to work outside of direct clinical practice.

## Approach

The Nursing and Midwifery Council (NMC) register contains information on everyone meeting required standards to practice in those professions in England and Wales (currently around 800 thousand people). Registrants must not only complete a course of study for registration but must revalidated every three years. The register has been linked to the 2021 census for England and Wales. The census includes questions ascertaining occupation. Information provided is categorised to provide indication of individuals’ main occupation. These include codes for nursing and for midwifery which provides indication not only to occupation but also to engagement in clinical practice. Analysis uses logistic regression to ascertain how socio-demographics influence engagement in clinical practice or otherwise.

## Conclusions and Implications

Data linkage for this study is now completed with the conference being the first time results will be shared. This will be the first time there has been concrete evidence as to nurses and midwives actual occupational roles. A stakeholder group supporting this work includes individuals from Chief Nurses’ Offices to enable findings to feed into policy.

